# Author Correction: Enhancement of electrocatalysis through magnetic field effects on mass transport

**DOI:** 10.1038/s41467-024-47977-z

**Published:** 2024-04-25

**Authors:** Priscila Vensaus, Yunchang Liang, Jean-Philippe Ansermet, Galo J. A. A. Soler-Illia, Magalí Lingenfelder

**Affiliations:** 1 Max Planck-EPFL Laboratory for Molecular Nanoscience and Technology, École Polytechnique Fédérale de Lausanne (EPFL), Lausanne, Switzerland; 2 Institute of Physics (IPHYS), École Polytechnique Fédérale de Lausanne (EPFL), Lausanne, Switzerland; 3 Instituto de Nanosistemas, Escuela de Bio y Nanotecnologías, Universidad Nacional de San Martín, San Martín, Buenos Aires, Argentina

**Keywords:** Electrocatalysis, Surfaces, interfaces and thin films, Energy, Materials for energy and catalysis

Correction to: *Nature Communications* 10.1038/s41467-024-46980-8, published online 3 April 2024

The original version of this Article contained an error in the schematic of Fig. 4a. The depiction of the magnetic field lines in Fig. 4a did not align with the description provided in the figure caption and the main text. Figure 4a is now redrawn and corrected in both the PDF and HTML versions of the Article.

