# Supplementary material for: Enhancement of electrocatalysis through magnetic field effects on mass transport
Source: Nat Commun. 2024 Apr 3;15:2867. doi: 10.1038/s41467-024-46980-8 (PMC10991325; doi:10.1038/s41467-024-46980-8)
Supplement: Supplementary file 1 — Supplementary Information [file 41467_2024_46980_MOESM1_ESM.pdf]

Enhancement of Electrocatalysis through Magnetic Field Effects on Mass Transport

Priscila Vensaus,[1,2,3,#] Yunchang Liang,[1,2,#] Jean-Philippe Ansermet,[2] Galo J. A. A. Soler-Illia,[3] Magalí Lingenfelder[1,2,*]

[1] Max Planck-EPFL Laboratory for Molecular Nanoscience and Technology, École Polytechnique Fédérale de Lausanne (EPFL), 1015 Lausanne, Switzerland.

[2] Institute of Physics (IPHYS), École Polytechnique Fédérale de Lausanne (EPFL), 1015 Lausanne, Switzerland.

[3] Instituto de Nanosistemas, Escuela de Bio y Nanotecnologías, Universidad Nacional de San Martín, San Martín, B1650 Buenos Aires, Argentina.

[#] These authors contributed equally in this work.

* maggie@lingenfelder-lab.com

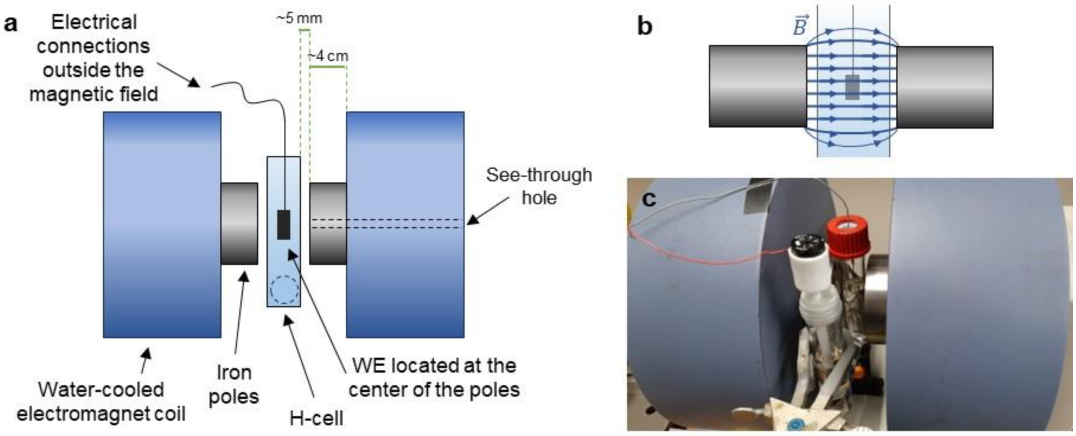

Supplementary Fig. 1 | Details of the magneto-electrochemistry setup. a, schematics of the electromagnet and the EC-cell, focused on the WE compartment. **b,** magnetic field lines surrounding the WE. **c,** photograph of the setup: the CE and RE are placed in the other H-cell compartment where the magnetic field is negligible, the H-cell is held in position by an aluminum (non-magnetic) holder.

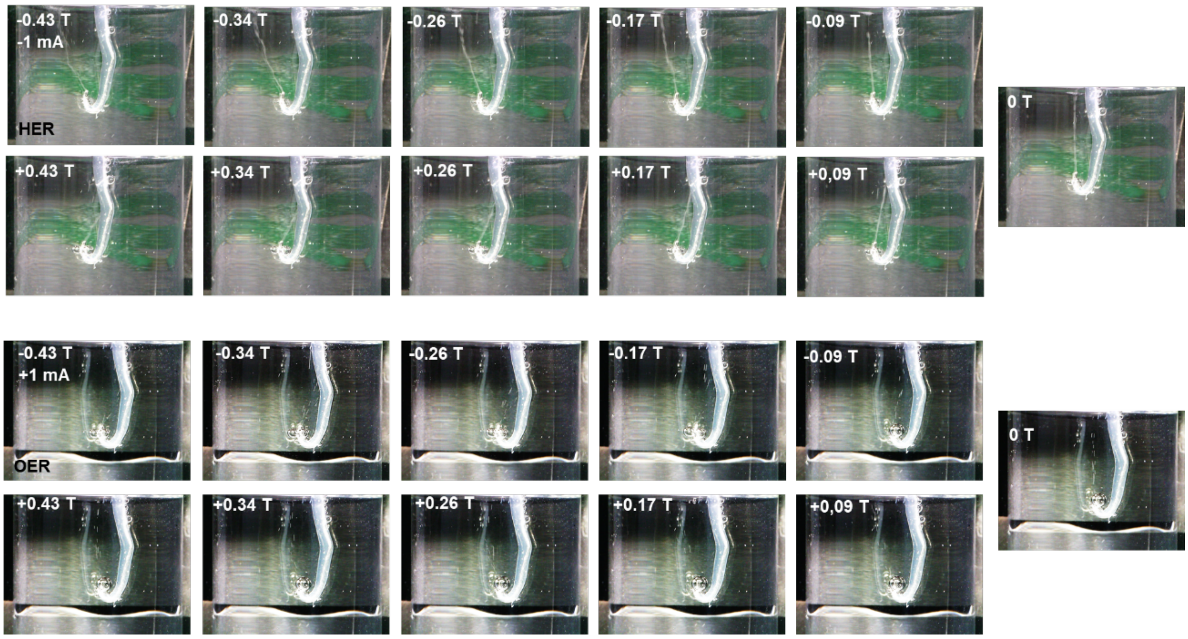

Supplementary Fig. 2 | Photographs of the H_2 and O_2 bubble movements under different magnetic fields. Photographs showing bubble formations at the tip of a wire covered with plastic isolation. The magnetic field is perpendicular to the plane of the picture. Top two rows: hydrogen evolution reaction (HER). The fine line emanating from the electrode is a tight string of small bubbles. The inclination of the string of bubbles changes sign when the field is reversed. Bottom two rows: oxygen evolution reaction (OER). Oxygen bubbles are larger and because they are separated from one another, a close inspection is needed to assess the effect of the magnetic field on their trajectory. For a given field, say -0.34 T, the hydrogen bubbles are deviated to the left (on the picture) and the oxygen bubble, to the right.

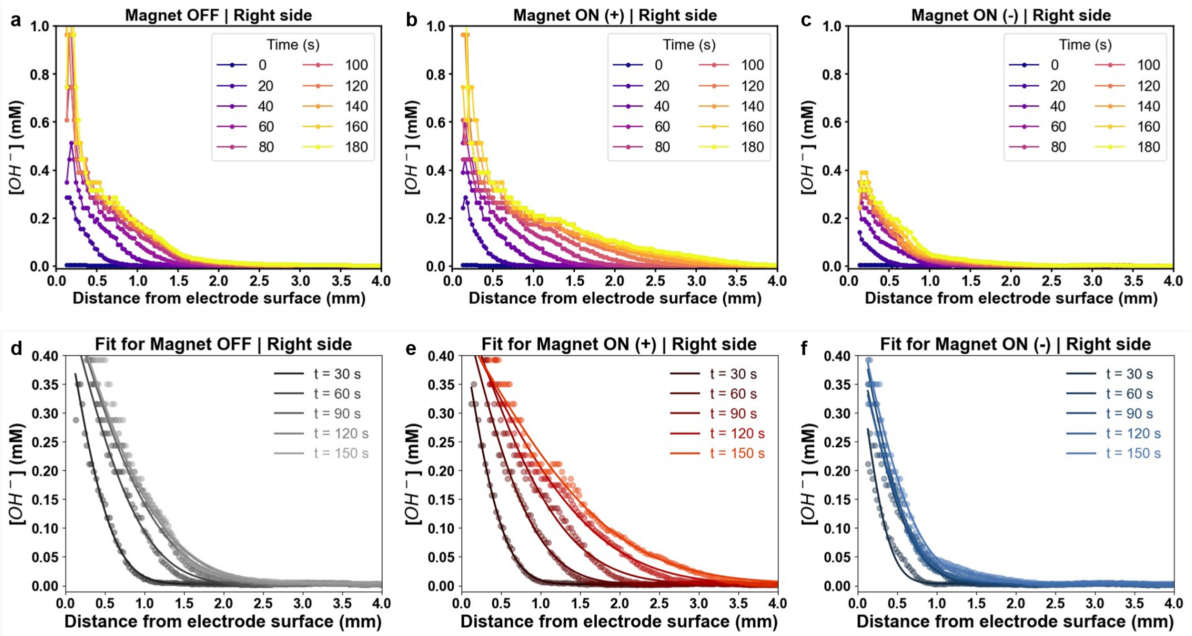

Supplementary Fig. 3 | OH^- concentrations as a function of time and distance. Estimated OH^- concentration profiles at the right side of the WE under different magnetic fields (a – c and d – f). Fits using eq. 7 and the data taken in the range of 0 to 0.40 mM (d – f).

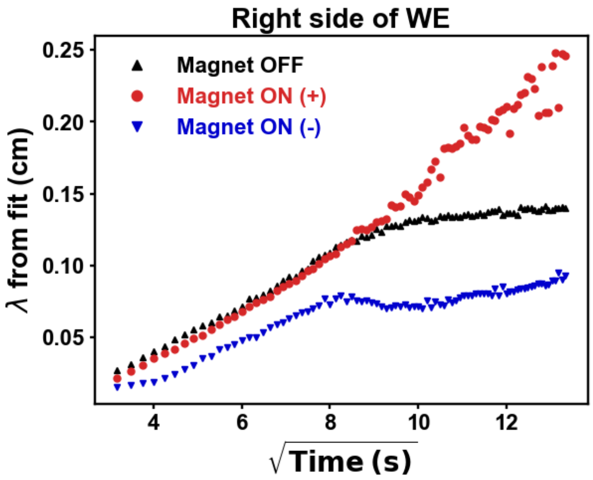

Supplementary Fig. 4 | OH$^-$ diffusion length (λ) vs the square root of time, estimated from the fitting of the concentration profiles shown in Supplementary Fig. 2. During the first 90 seconds of the reaction, the diffusion length follows a linear relationship with the square root of the time, as described by fickian diffusion ($\lambda = 2\sqrt{Dt}$). After this time, diffusion of hydronium ions towards the right side of the Pt electrode seems to increase when the magnetic field is turned on towards the positive direction (slope slightly increases), while it leans towards a plateau in the case of negative direction or when the magnetic field was turned off.

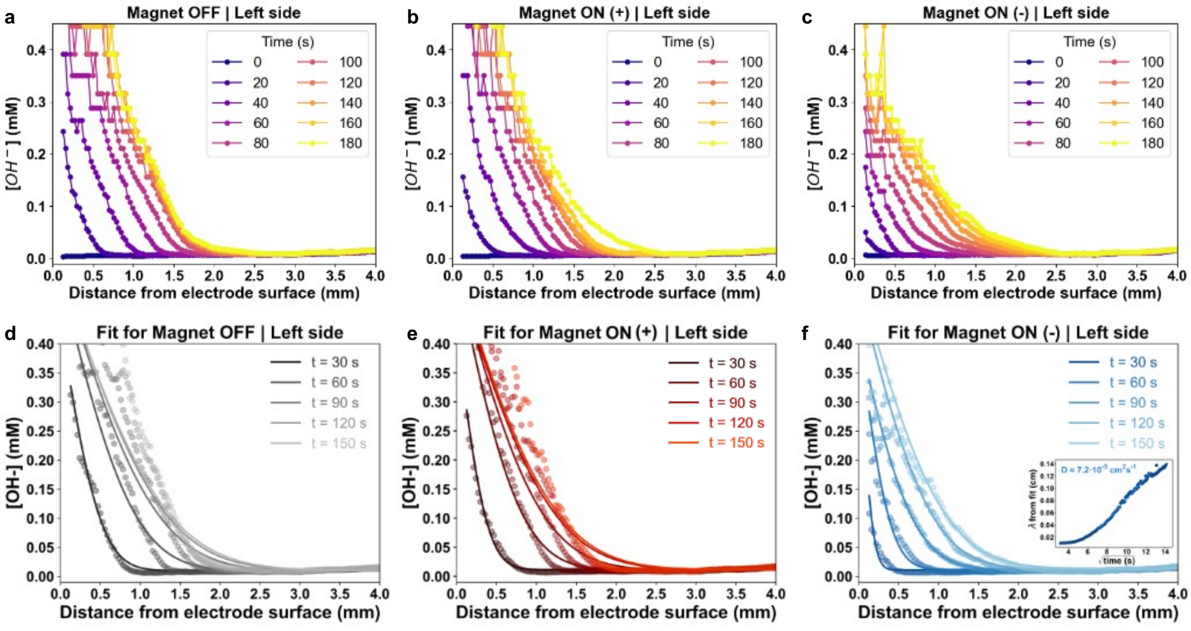

Supplementary Fig. 5 | Estimated OH$^-$ concentration profiles at the left side of the WE under different magnetic conditions (a – c) and their corresponding fits with equ 7 for data in the range of 0 to 0.40 mM (d-f). Here, the OH$^-$ concentration extends further in the magnet ON (-) condition than when the magnetic field was turned off or in the other direction (i.e. with the magnetic field ON (-) there is a higher OH$^-$ concentration beyond 2 mm away from the electrode surface at 180 s compared to the magnetic field off). This indicates a faster diffusion towards the left side in this case. Good fits using eq. 7 were obtained only with negative magnetic fields. The inset shows fickian behavior with a diffusion constant *D* of $7.2 \cdot 10^{-5}$ cm^2 s^{-1}, a value higher than that obtained in Fig. 4 for the condition without magnetic field. The lack of good fits in the other cases was most likely caused by the shadows posed by the WE as the light was shone from the right side, making it more difficult to differentiate

the indicator from the background with the software and thus a less accurate estimation of OH^- concentration.

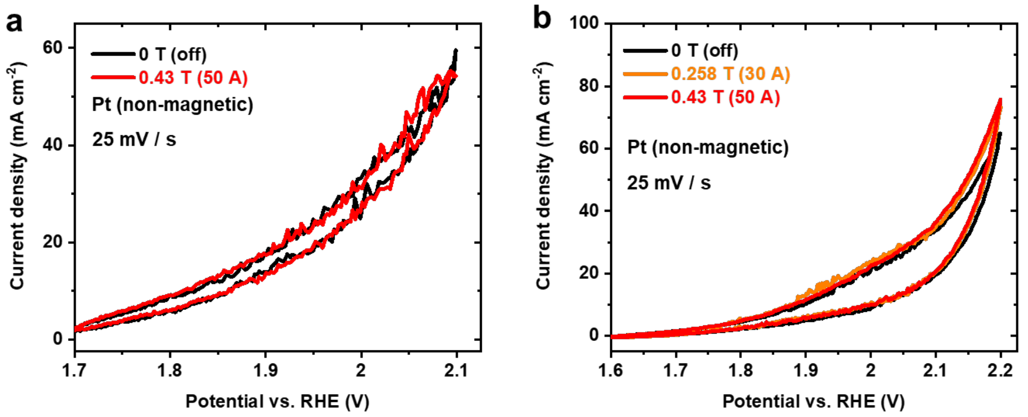

Supplementary Fig. 6 | OER at a Pt microelectrode. **a,** No magnetic field effect can be seen when the upper potential is 2.1 V vs. RHE. **b,** Higher overpotentials leads to enhancement in the current when a magnetic field is applied.

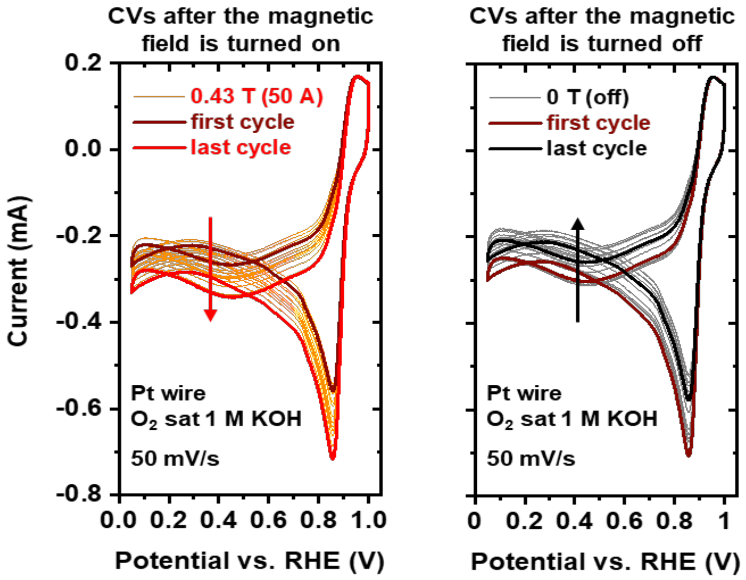

Supplementary Fig. 7 | CV of a Pt wire in O_2-saturated KOH. The magnetic field was first switched to 0.43 T and several scans were recorded. Then the magnetic field was turned off. The first and last cycles in both cases are highlighted in bold. When the magnetic field was turned on, the Lorentz-force induced stirring facilitates the movement of O_2 toward the WE. When the magnetic field is turned off, O_2 gets consumed at the WE and the limiting current is reduced.

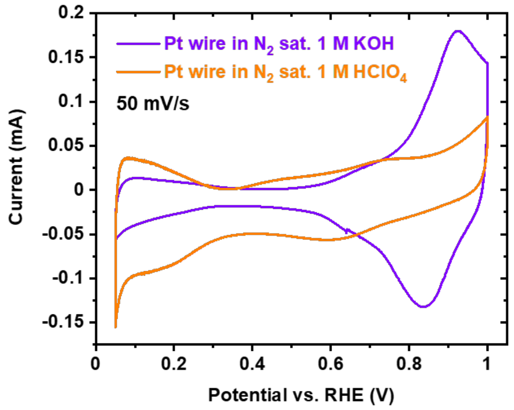

Supplementary Fig. 8 | CVs of the Pt wire in N_2 saturated electrolytes. Typical CVs of Pt wire in N_2 saturated KOH and $HClO_4$. Note that the surface of the Pt wire under the electrolyte level is different in different experiments.

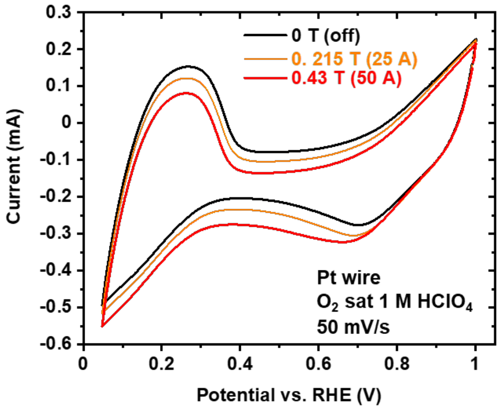

Supplementary Fig. 9 | CVs of a Pt wire in O_2-saturated $HClO_4$. The magnetic field was first switched from 0 T (off) to 0.215 T, and finally, to 0.43 T. Only the last cycles under each magnetic field are shown here.

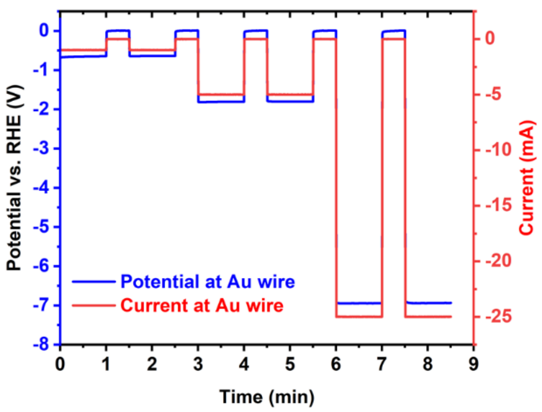

Supplementary Fig. 10 | Potential and current at the Au WE next to the isolated Pt wire.

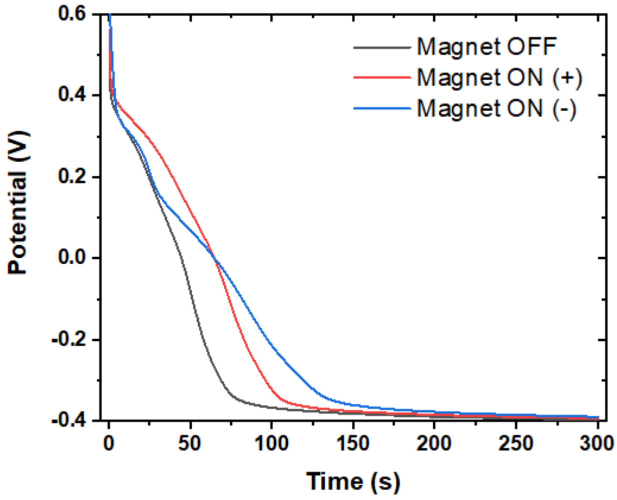

Supplementary Fig. 11 | Chronopotentiometries at -0.2 mA of Pt foil WE during the recording of Supplementary Video 3.

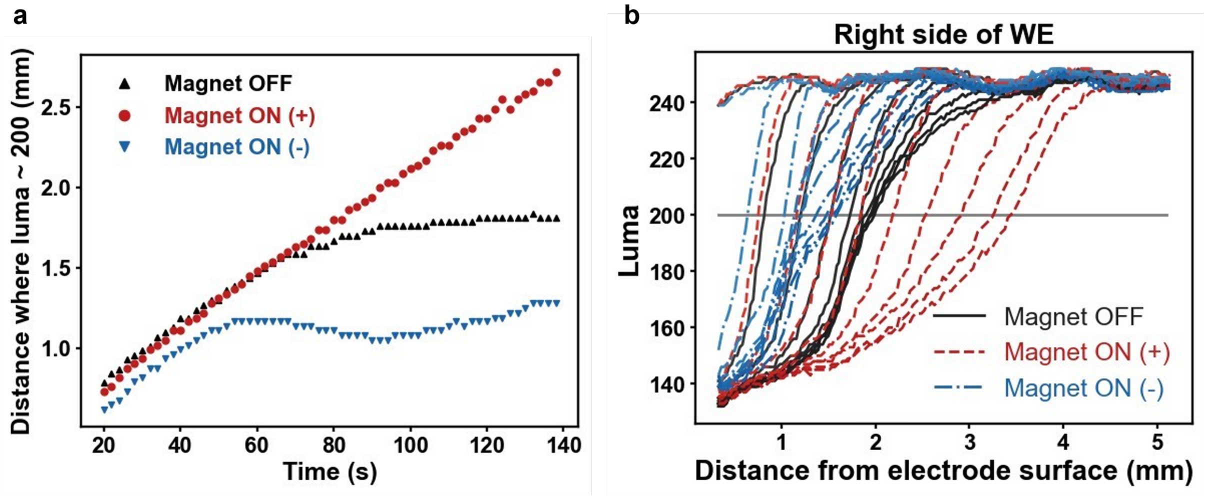

Supplementary Fig. 12 | a, OH^- diffusion length (λ) vs. time, estimated by taking the value where luma (brightness) = 200 in b.
