# Supplementary material for: Enhancement of electrocatalysis through magnetic field effects on mass transport
Source: Nat Commun. 2024 Apr 3;15:2867. doi: 10.1038/s41467-024-46980-8 (PMC10991325; doi:10.1038/s41467-024-46980-8)
Supplement: Supplementary file 3 — Description of Additional Supplementary Files [file 41467_2024_46980_MOESM3_ESM.pdf]

Description of Additional Supplementary Files

Supplementary Movie Legends

Supplementary Movie 1: Videos of the H2 (left) and O2 (right) bubble stream movement under different magnetic fields.

Supplementary Movie 2: OER on a Pt mesh during CV under an applied magnetic field of -0.43 T. The produced oxygen bubbles follow a whirling motion when the potential is applied under the magnetic field.

Supplementary Movie 3: OER on a Pt mesh during chronopotentiometry at 20 mA, under applied magnetic field. The magnetic field was changed from 0.43 T to -0.43 T and back. The produced oxygen bubbles follow a whirling motion when the magnetic field is applied and the CP is running. The direction of the bubbles displacement is dependent on the strength and direction of the magnetic field. Once the CP was stopped (timestamp ca. 1:11:75), the bubbles follow their natural upward motion.

Supplementary Movie 4: OER on a Pt mesh during CV with a strong permanent magnet located below the electrochemical cell. The produced oxygen bubbles follow an upwards whirling motion.

Supplementary Movie 5: Oxygen bubbles produced by hydrogen peroxide decomposition at a Pt wire electrode, not connected to the potentiostat, in the vicinity of a gold coil WE (not seen; see Figure 3b for details). Different magnetic fields and applied currents to the gold are shown. The direction of movement of the stream of bubbles is affected by the applied current and the intensity of the magnetic field. Turning off the reaction (current = 0 mA) leads to a natural movement of the bubbles towards the top of the cell.

Supplementary Movie 6: Observation of OH- accumulation and movement around a Pt foil electrode using phenolphthalein indicator during ORR, with an applied current of - 0.2 mA, a) with magnetic field turned off, b-c) turned on in opposite directions with 0.43 T.

Supplementary Movie 7: Observation of OH- accumulation and movement around a Pt foil electrode using phenolphthalein indicator during ORR, with an applied current of - 0.4 mA, and its subsequent oxidation (OER) with an applied current of 0.4 mA. a) with magnetic field turned off, b-c) turned on in opposite directions with 0.43 T.
